# Granulosa tumor: two spontaneous pregnancies after combined medico-surgical treatment: case report and review of the literature

**DOI:** 10.1186/s13256-023-03793-5

**Published:** 2023-03-30

**Authors:** Mohamed Kaakoua, Jean Chidiac, Andrianandrasana Notf, Ruiqian Chen, Isabelle Mahe, Sadji Djennaoui

**Affiliations:** 1 Hôpital Louis Mourier (Colombes) -AP-HP, Nord-Université de Paris Cité, Colombes, France; 2 Hôpital Bichat - Claude Bernard (Paris) -AP-HP, Nord-Université de Paris Cité, Paris, France

**Keywords:** Obstetric prognosis, Granulosa tumor, Therapeutic, Pregnancy, Case report

## Abstract

**Background:**

Granulosa tumor is a rare tumor that arises from the mesenchyme and the sexual cord of the ovary. The prognosis is generally excellent, and treatment is mainly based on surgery, followed by chemotherapy depending on the extension of the disease. However, “the obstetrical prognosis” is compromised.

**Case presentation:**

We report the case of a 32-year-old Caucasian patient who was diagnosed during a primary infertility assessment with an ultrasound image of a 39 mm organic left ovarian cyst confirmed on pelvic magnetic resonance imaging with infiltration of the uterosacral space. Tumor markers, including cancer antigen 125, alpha fetoprotein, and β-human chorionic gonadotropin, were normal. Histological study of biopsies of the ovarian lesion taken during exploratory laparoscopy confirmed the diagnosis of adult granulosa tumor. After a normal extension assessment including a thoracoabdominopelvic computed tomography scan and a positron emission tomography scan, the patient underwent complete conservative surgery and the disease was classified as stage Ic. Three cycles of adjuvant chemotherapy according to the “BEP” protocol combining bleomycin, etoposide, and cisplatin were performed after oocyte cryopreservation. After a 5-year follow-up period, the patient had no sign of tumor progression and had two spontaneous pregnancies, the first occurring 3 months after the end of chemotherapy and the second 14 months later.

**Conclusion:**

Granulosa cell tumor remains a rare tumor whose management considerably compromises fertility and reduces the chances of having a spontaneous pregnancy. The particularity of our observation is that the diagnosis of the granulosa tumor was made following a primary infertility assessment and that the patient had two spontaneous pregnancies 3 months after the end of a medico-surgical treatment known to be very gonadotoxic.

## Background

Granulosa tumor (GT) is a rare entity of ovarian malignant tumor developed from stromal cells and the sex cord [1]. GT is associated with a very favorable prognosis, especially at the localized stage thanks to surgery, which remains the standard treatment, with or without adjuvant chemotherapy, depending on the stage and the prognostic factors of the disease [2, 3]. All these therapies compromise the fertility of young patients wishing to become pregnant. Nevertheless, the preservation of fertility by oocyte cryopreservation remains an option offered to these patients before any treatment [4]. The possibility of spontaneous pregnancy after combined medico-surgical treatment of ovarian granulosa tumor remains a matter of great concern.

We report the observation of a patient with this tumor, and discuss through this observation the implication of this tumor and treatment on the obstetrical prognosis.

## Case presentation

**Fig. 1:**
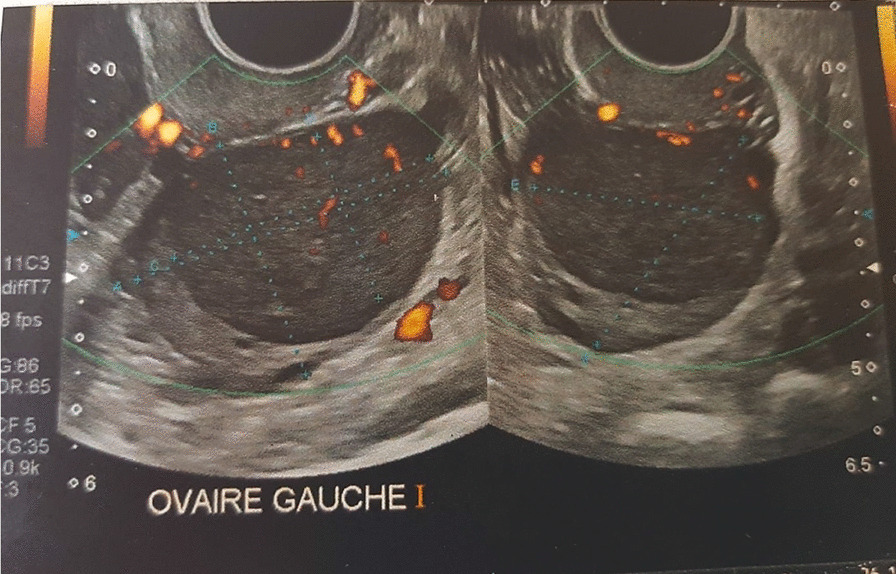
Ultrasound image showing an organic left ovarian mass measuring 39 mm

**Fig. 2:**
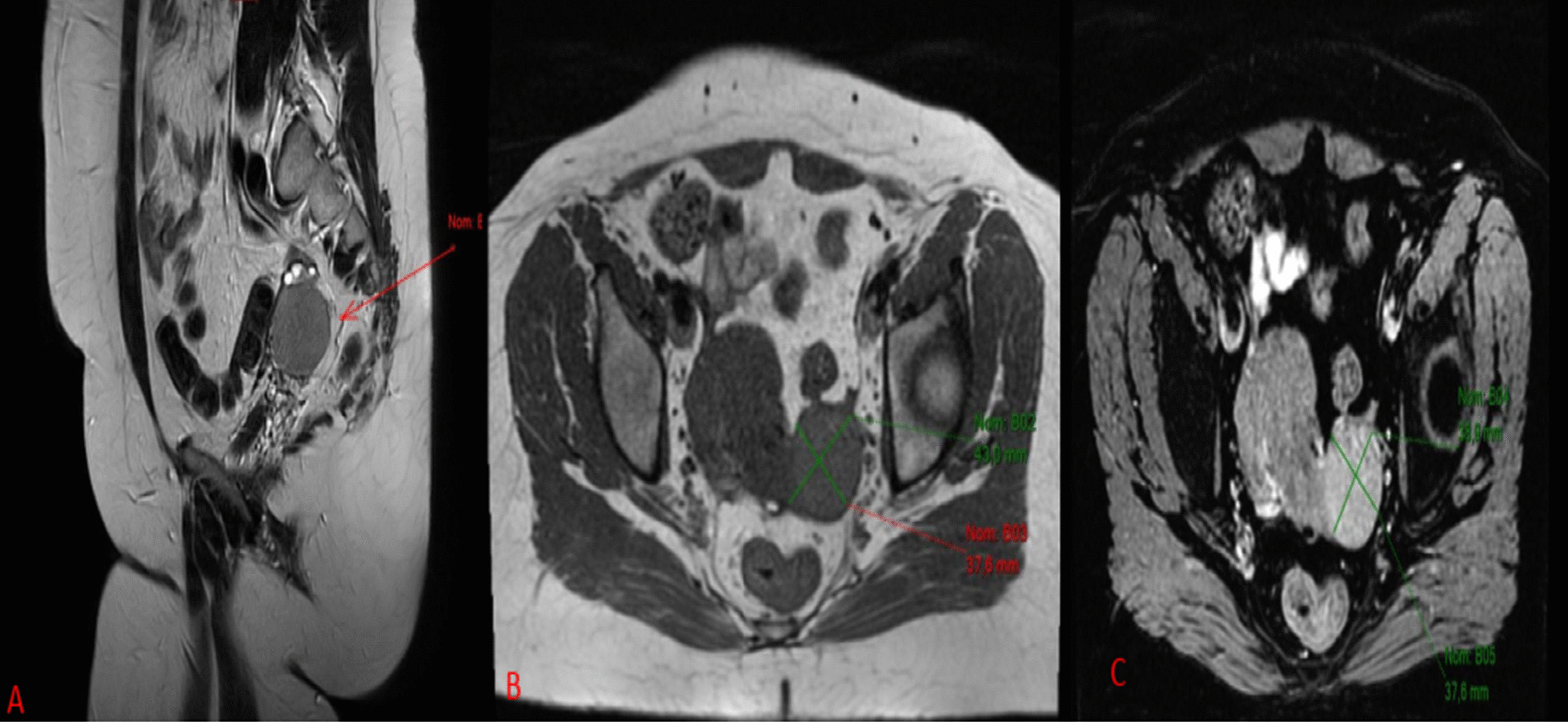
Pelvic MRI showing the presence of a left ovarian mass. **A** Sagittal slice in T1 sequence, **B** axial slice in T1 sequence, **C** axial slice after injection of gadolinium

**Fig. 3:**
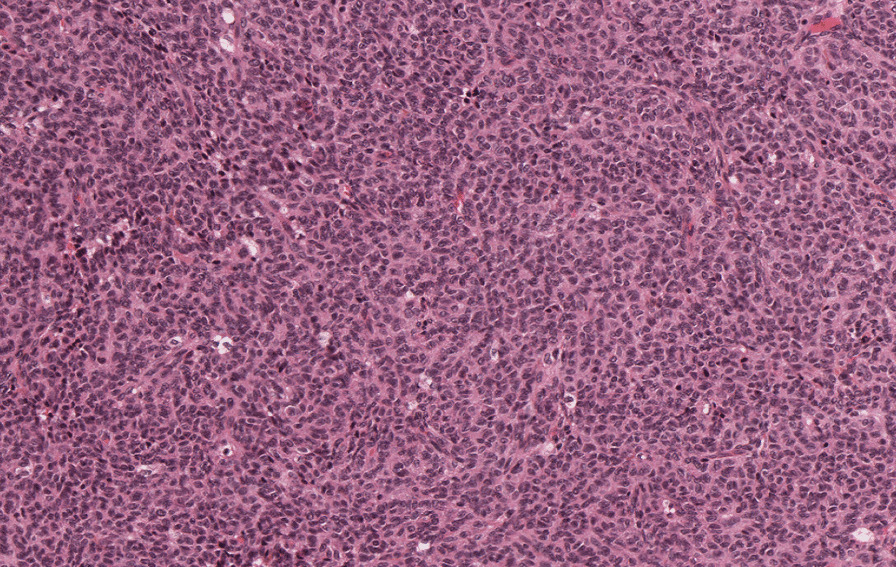
Tumor proliferation of diffuse and microfollicular architecture (×100)

**Fig. 4:**
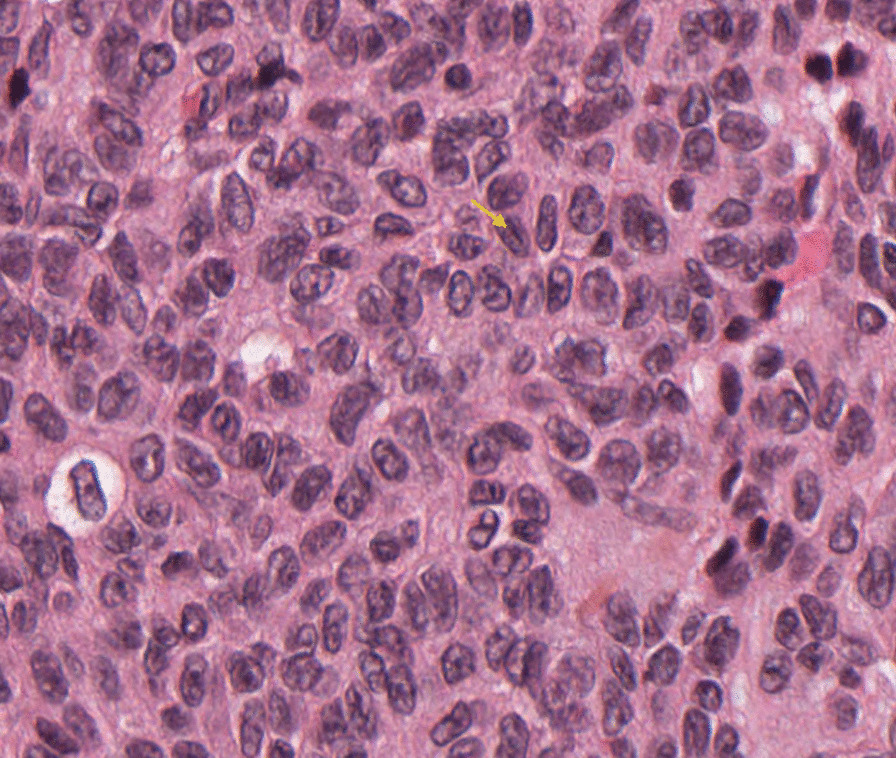
Cell with central notch, coffee bean (×400). Arrow corresponds to the coffee bean pattern in granulosa cell tumors

We report the case of a 32-year-old patient, of Caucasian origin, with no notable medical history, nulliparous and nulligravida, who suffered from primary infertility for more than 2 years and in whom, during a primary infertility assessment, a 39 mm left ovarian cyst of organic appearance was observed on pelvic ultrasound (Fig. 1). Pelvic magnetic resonance imaging (MRI) confirmed the presence of a left ovarian mass with infiltration of the uterosacral space (Fig. 2). Biologically, ovarian tumor markers including cancer antigen 125 (CA 125), inhibin B, α-fetoprotein, and β-human chorionic gonadotropin (β-HCG) were all normal, as well as ovarian hormone assessment, pituitary hormones [follicle-stimulating hormone (FSH) and luteinizing hormone (LH)], and anti-Müllerian hormone (AMH) level. Regarding these clinical, radiological, and biological elements, an exploratory laparoscopy was performed and revealed an enlarged left ovary with a dystrophic parenchyma. Multiple biopsies were performed and showed a morphological and immunohistochemical aspect of granulosa tumor of the adults (Figs. 3 and 4). The exploration was completed by hysteroscopy and an endometriectomy. Histology was normal, with no signs of hyperplasia or malignancy. As part of the extension assessment, chest, abdominal, and pelvic computed tomography (CT) scan and positron emission tomography (PET) scan were requested, which showed no abnormality except the known left ovarian cyst. The multidisciplinary oncogynecology tumor board recommended conservative surgery including left anexectomy and infra-colonic omentectomy with peritoneal biopsies, and peritoneal cytology followed by three cycles of adjuvant chemotherapy every 3 weeks according to the “BEP” protocol combining bleomycin (30 units intravenously, days 1, 8, 15), etoposide (100 mg/m^2^, days 1–5), and cisplatin (20 mg/m^2^, days 1–5) was decided at the multidisciplinary tumor board, preceded by oocyte cryopreservation. The definitive histology on the surgical specimen confirmed the laparoscopic exploration data. Cytology showed slightly hematic peritoneal fluid, evoking perioperative rupture. The disease is classified as stage Ic1. After 9 weeks of chemotherapy, a check-up including thoracic, abdominal, and pelvic scans as well as tumor markers, in particular HCG, was normal. Subsequently, the patient had two spontaneous pregnancies that proceeded to term, with vaginal delivery, the first of which occurred 3 months after the end of the chemotherapy and the second 14 months later. After 5 years of follow-up, the patient has no sign of tumor progression and the two children are in good health.

## Discussion

Granulosa cell tumors are malignant neoplastic diseases that belong to the group of mesenchymal and sex cord tumors of the ovary [5]. This group makes up approximately 3–5% of ovarian cancers. However, GTs account for more than 70% of tumors in this group [6]. It is a rare entity whose incidence varies between 0.58 and 1.6 cases per 100,000 patients [7]. These tumors can occur at any age, but the adult form remains the most common one, occurring most often during perimenopause (95%). It can also occur earlier in young women under 30 years old or in the prepubertal period for the juvenile form, which accounts for 5% of these tumors [8].

Clinically, granulosa tumors can manifest as a tumor syndrome associated with abdominal and pelvic pain and/or an endocrine syndrome in case of estrogen hypersecretion in the form of precocious pseudo-puberty in young women, metrorrhagia and endometrial hyperplasia in postmenopausal women, or hirsutism and clitoral hypertrophy in the event of androgenic secretion [9, 10]. The clinical presentation of infertility, in particular anovulatory infertility, is often associated with GT in women of childbearing age [11]. In our case, the diagnosis of adult granulosa tumor was made during a primary sterility exploration. The radiological appearance of granulosa tumors is not unequivocal. It is generally a latero-uterin, cystic, or solidocystic mass, often large, hypervascularized, and without vegetations [12]. Faced with this great morphological variability, the presence of some serum markers can lead to the diagnosis of granulosa tumors, in particular inhibin B and anti-Müllerian hormone (AMH) [13]. Definitive diagnosis of granulosa cell tumors is made by histological description by highlighting uniform cell clusters with a coffee bean appearance of the nuclei that may surround eosinophilic bodies to form Call–Exner bodies (which are pathognomonic but inconstant). The expression of some markers by immunohistochemistry, in particular vimentin, calretinin, and α-inhibin, as well as the search for molecular abnormalities such as the FOXL2 402C-G mutation make the diagnosis of these tumors easier [14, 15]. The management of granulosa tumors depends on the initial extension of the disease and the desire for pregnancy. For localized stages, surgery remains the cornerstone. It includes an exploration step and a complete resection step that can be conservative or radical. Adjuvant therapy treatment is sometimes indicated, especially from stage Ic onward [3]. In general, granulosa cell tumors have a good prognosis because they are usually of low-grade malignancy. The 10-year survival rate varies between 84% and 95% for stage I, 50% and 65% for stage II, and 17% and 33% for stage III/IV [16]. Our patient is still in complete remission after 5 years of follow-up.

Although these tumors have excellent prognosis after initial treatment, complications may occur such as high risk of infertility in women of childbearing age. All the therapies recommended in the management of these tumors cause toxicity and hormonal complications, particularly gonadal, with adnexal surgery leading to a decrease in follicular reserves [17]. This risk is all the greater if the surgery is followed by gonadotoxic adjuvant. The incidence of spontaneous pregnancy just after combined and multimodal treatment of granulosa tumor is not well elucidated. In a large retrospective series of 146 patients followed for granulosa tumor reported by Unkila-Kallio *et al.* [11], only 5 patients achieved at least one pregnancy after surgery. In two other large series of 113 patients reported by Lee *et al.* [24] and Wang *et al.* [27], the incidence of pregnancy after treatment was 14.1% and 17.6% of cases, respectively. However, Wang *et al.* [28] reported a series of 35 patients who all received surgical treatment and more than 88% of them adjuvant chemotherapy. Only six patients had a spontaneous pregnancy after this multimodal treatment (17.1%). Table 1 summarizes the different cases reported in the literature of spontaneous pregnancy after treatment of a granulosa tumor.

**Table 1:** Pregnancies after treatment of granulosa cell tumor reported in the different series in the literature

Authors and year of publication	Granulosa tumor (*n*)	Conservative surgery	Adjuvant chemotherapy	Post-treatment pregnancies
Piura *et al.* 1994 [23]	18	18/18 (100%)	Not mentioned	3/18 (16.6%)
Unkila-Kallio *et al.* 2000 [11]	146	Not specified	Not mentioned	5/146 (3.4%)
Lee *et al.* 2011 [24]	113	46/113 (40.7%)	35/113 (30.9%)	16/113 (14.1%)
A. Karalok *et al.* 2015 [25]	10	10/10 (100%)	2/10 (20%)	5/10 (50%)
N. Rinne *et al.* 2017 [26]	2	2/2 (100%)	Not indicated	2/2 (100%)
D. Wang *et al.* 2018 [27]	113	61/113 (53.9%)	48/113 (42.47%)	20/113 (17.6%)
D. Wang *et al.* 2021 [28]	35	35/35 (100%)	31/35 (88.5%)	6/35 (17.1%)

Gonadal resistance to conventional therapy depends mostly on the mechanism of action of the molecules, the doses administered, and the ovarian follicular potential at the time the molecules are administered [18]. Among the various regeneration molecules, the alkylating agents, including platinum salts, are those whose gonadotoxic effect is the best documented and the most pronounced [19]. The gonadotoxic potential of cytotoxic therapies is classified into three categories of infertility risk. A therapy is considered to have high gonadotoxic potential if the risk is greater than 80%. This risk is intermediate if it is between 40% and 60% and low if it is less than 20% [20]. Our patient received adjuvant chemotherapy according to the BEP protocol, which is considered to have intermediate gonadotoxic potential [21] (Table 2).

**Table 2:** Risk of infertility in women according to chemotherapy drugs [29]

Risk of infertility	Chemotherapy drugs
High risk (> 80% permanent amenorrhea)	Alkylating agents Nitrogen mustard: cyclophosphamide (≥ 75 g/m^2^, women < 20 years old), ifosfamide, melphalan, chlorambucil Methanesulfonate: busulfan Triazine: dacarbazine, procarbazine
Moderate risk (40–60% permanent amenorrhea)	Alkylating agents Platinum analog: cisplatin, carboplatin Nitrogen mustard: cyclophosphamide (woman 29–30 years old) Anthracycline: doxorubicin, epirubicin Moderate risk protocols BEP: bleomycin, etoposide, and cisplatin
Low risk (< 20% permanent amenorrhea)	Vinca alkaloids: vincristine, vinblastine Antimetabolite: mercaptopurine, methotrexate Cytotoxic antibiotic: bleomycin and actinomycin D
Very low risk or no risk	Taxanes: docetaxel, paclitaxel Antimetabolite: cytarabine

The recovery of gonadal function depends on several factors, including the treatment protocol, the duration of treatment, and the age of the patient. The time to recovery of this function after granulosa tumor treatments has not been reported in the literature. However, recovery of this function in series of patients treated with chemotherapy for breast cancer generally occurred within 6–12 months after the end of treatment [22].

The particularity of our observation is that the pregnancy occurred in a patient with primary sterility, only 3 months after presumed gonadotoxic medical and surgical treatment.

## Conclusion

Granulosa cell tumor is a rare tumor, but has a good prognosis. However, its treatment significantly compromises fertility. Spontaneous pregnancy just after the end of the medico-surgical treatment remains exceptional, especially if the patient already has a primary sterility. Nevertheless, it is strongly recommended to refer patients, before each gonadotoxic treatment, to a specialized reproductive medicine consultation to assess the gonadotoxic risk associated with the treatments and to discuss the most appropriate means of preserving the fertility of these patients.

## Data Availability

Data sharing not applicable to this article as no datasets were generated or analyzed during the current study.
